# Diseases of the Mouth

**Published:** 1861-07

**Authors:** Jas. E. Garretson

**Affiliations:** Philadelpeia


Diseases of the Mouth.—*Anomalous Tumors.*—By *Jas. E. Garretson, M. D., of Philadelpeia.*—Not unfrequently there may be seen standing, isolated and alone, on some portion of the dental arch, most frequently, however, either on the anterior portion of the interior arch, or the posterior portion of the superior, a yellowish-looking tumor, which might not inaptly be compared to a shell-bark covered with inspissated mucus. Sometimes this tumor will be found quite firm in its position, seeming, indeed, as if it might have sprung from the socket of some long ago extracted tooth; at other times you will be able to move it quite freely, as if it had fleshy peduncles. These tumors give to the patient a most disgusting appearance, are insufferably offensive, and so detrimental to health, that five or six grains of their substance, given to a small animal, will not unfrequently cause its death. The composition of such tumor consists of phosphate and carbonate of lime, epithelial scales, inspissated mucus, and the various debris of a cavity devoted to mastication. In other words, they are salivary calculi. The nucleus of such a growth is, of course, a tooth. The manner of formation is too evident to need description. I have removed these calculi, where the nucleus had become so encysted, from crown to apex, that no trace of it was to be discovered without dividing the mass. Where, however, the encystment has advanced to this extent, the tumor is about ready to drop from the mouth of its own accord.

I have seen a calculus of this kind encyst the six lower front teeth, making as strange a looking tumor as could be well imagined.

Similar calculi develop, as may be inferred from my last paper, in other parts of the mouth. Thus, just within the orifice of the duct of Steno they may, occasionally, be found; the tumor, in such a case, bulging out from the cheek against the second molar tooth of the upper jaw. The formation of such tumors, in these situations, does not necessarily imply the closure of the orifice of the duct, but they form when the gland is sluggish, the secretion not being in sufficient abundance to hold the lime of the saliva in solution until it is ejected from the duct, it thus falls on the flow of the duct, and, lodging, makes the nucleus.

I remember, on one occasion, to have been called by a fellow practitioner to see a case where a mass of this calcareous matter, quite the size of the largest almond, seemed to be growing from all that portion of the sublingual region anterior to the gland of that name ; one-half the tumor looked as if it might be below the level of the floor of the mouth, the mucous membrane enveloping the mass with ragged and ulcerated edges. It certainly presented a very strange and threatening look. My friend was deceived as to its character, because there was no apparent direct association between the tumor and the neighboring teeth ; and because it was as firmly fixed as though it might have been a growth springing from the neighboring bone.

But yet it was a salivary calculus, and nothing else, the only question being as to its cause and fixedness.

Looking about the mouth, I perceived that the patient had certain artificial teeth on the left side of the arch, these teeth were all coated with tartar—salivary calculus—and so associated thereby with the natural teeth as to be only distinguishable by that difference in the translucency so immediately noticeable by any one experienced in such a direction. Knowing well that it is a plan with many dentists to secure such teeth by passing a strong gold wire across the mouth, and which wire not unfrequently buries itself within the mucous membrane, thereby occasionally concealing it, I inferred at once that such a wire would be found the nucleus of our calculus, and directed an examination accordingly. This examination was commenced by cutting away the calculi from about the artificial teeth, and, as anticipated, the band of wire was revealed ; next was sought the concealed attachment of the opposite side, and this being discovered and exposed, the two ends were forced from the teeth which they clasped, and thus the artificial teeth, wire, and calculus were lifted from the mouth in a body.

The site of the calculus, as may be inferred, presented a cup-shaped ulcerated depression, and was quite angry looking.

The only after treatment consisted in the use of an astringent wash. The ulceration healed kindly in a very few days.

I may be allowed to suggest to the physician, that the existence of such calculi is not an unfrequent cause of dyspeptic and other alimentary troubles. I have just now, in my mind, a case of dyspeptic consumption, of which, truly, it might have been said "physicians were in vain," the patient having tried every class, from the professor down to the quack.

In her mouth she had but a single tooth, and this for years had been so imbedded in salivary calculus, as much more to resemble the half rotted shell bark than a tooth—her breath was made insufferable by it. I removed the offensive mass, and the recovery of the patient was really magical in its rapidity.

Such calculi are to be removed in any convenient manner; they may be pulled away, broken away, or, when loose, may be cut from the gum; the operation being entirely a mechanical one.

I forget, however, in such advice, my reference to calculi situated in the duct of Steno. These are to be removed, either by enlarging the duct and crushing the stone, or otherwise by cutting down upon it at the most convenient point. When so cut down upon, the wound will not commonly require any after attention.

There is another class of anomalous tumors sometimes to be met with in the mouth, and which, of all diseases of this cavity, are, perhaps, most simple, and yet most often misunderstood. I allude to fungoid excrescences from the tooth pulp. These tumors, frequently misnamed epules, fungus hæmatodes, etc., grow most commonly from cavities in the molar teeth. When not overhanging the walls of the tooth, their nature is, at a glance, of course appreciated; but sometimes the growth is so extensive as not only to overhang the tooth, but to completely conceal it, the morbid mass moulding itself over the gum. Here it simulates epulis; yet the practitioner will not be deceived if he remembers that epulis is a growth from the periosteum, while the mass can be lifted from the gum at any point of its circumference.

Its likeness to fungus hæmatodes consists alone in its external features, certainly not at all in its history. Such tumors, bleeding, not unfrequently, at the slightest touch, while they possess the *noli me tangere* quality, the result of the constant irritation to which their situation necessarily subjects them. These tumors are wholly benign, and perfectly amenable to treatment.

The reader has, without doubt, seen a carious tooth, the cavity of which was, more or less, filled with a fleshy growth. This is the pathology of the tumor I have described.

Such a growth may, perhaps, be observed to exist in one out of five hundred decayed teeth, certainly it is not more frequent than this, particularly if we except the deciduous teeth; but the expansion of this fungus, so as to cover the tooth, is still so much more rare, that there are, I imagine, few practitioners who have ever had the opportunity to see the condition.

The dental practitioner, who is the one most apt to be consulted in the more limited form of the affection (that is, where the fungus is confined to the cavity of the tooth,) treats it most successfully by an application of the arsenical paste—the formula of which I gave in the paper on "Anomalies." A portion of this paste, say a piece the size of a pin's head, is laid directly on the tumor, and being covered in with wax, or a pledget of cotton, is allowed to remain from twenty-four to forty-eight hours. After the lapse of this time—the vitality of the growth being destroyed—the mass, including all that pertains of the pulp which is enclosed within the fangs, may be easily aud painlessly removed. The diseased tooth, after a little medication, may not unfrequently be plugged and made useful for a considerable time.

When, however, the disease has so progressed that the tumor overhangs the tooth, let the remedy be extraction. Now, although I am not unaware that such extraction is denounced as highly dangerous by authors who have written on the subject—a fear of unconquerable hemorrhage being held up for the staying of the hand of the operator—yet I feel free to pronounce, that any surgeon who has so written has never practically known the disease.

It certainly is true, that these tumors, when in situ, not unfrequently bleed to an enormous extent on the slightest provocation, but it is not to be inferred from such bleeding, that the artery of the tooth is not in a healthy state just outside the foramen, or that it does not possess an amount of contractile force equal to that of any of its neighbors. When the tumor is cut, it has no power to arrest hemorrhage, because, from its fungoid character, it is almost entirely deficient in vital force. The blood which is being passed in it through its arterial supply, may be said to drain away, just indeed as it would drain through a sieve until arrested through the mechanical influence of a clot which would be apt finally to form about the meshes of the wire.

Such authors as I make reference to, recommend the use of caustics and ligatures.

As above, I recommend in these bad cases the extraction of the tooth. You had better have nothing to do with trying to save such teeth—there is a bad cachexia about them.

If there should be any one who, acting on my advice, finds that he has brought this trouble of hemorrhage on himself, he will find that he is entire master, by adopting the following simple manipulation:

Take a common cork, and make in it a V shaped cut. Next, procuring the cellar cobweb, saturate it with some tonic astringent,—the cinchona rubra answers admirably,—stuff this into the bleeding alveolus, and placing the V cut in the cork saddle-wise, over the gum, hold it firmly in place by binding the gums together by means of a Barton's bandage.

En passant.—This little manipulation, if properly conducted, will place all hemorrhage from the alveoli of teeth completely under the control of the surgeon. The after-treatment consists simply in leaving the clot to nature.

Very ugly, epulic-looking tumors are sometimes formed, the result of an absorption of the alveolar process from about the roots of the teeth. A sufficiently illustrative example is recorded by Dr. Suesserott, of Chambersburg.

The Doctor informed me, in the course of a conversation I had with him, that on one occasion, being in the alms-house of a neighboring county, he was invited by the attending physician to look at a case of very rapidly advancing cancer in the mouth of one of the women patients.

The tumor, as described by the Doctor, must have looked quite threatening enough. It occupied all the alveoli labial space, bounded by the canine teeth of either side, and overhung the teeth which lay posterior to it. It was very much ulcerated, dark and turgid, and bled freely on the slightest provocation. The diagnosis was fungus hæmatodes.

Curiosity prompting Dr. S. to push the fungoid mass back from off the teeth, that he might thereby examine its base, he was satisfactorily surprised to find projecting into the very middle of the tumor the roots of the incisor, from over which the process had been absorbed.

Convinced that here was the primary lesion, he suggested the immediate extraction of these diseased teeth. The advice was acted on. The tumor, at once, began to diminish in size and formidableness, and in a very few weeks every trace of it had disappeared.

Mr. Smith, from whose clinical practice I have before quoted, gives the following, in part, parallel case:

"Elizabeth H., aged forty, was sent from some distance in the country to the infirmary, Dec. 12, 1856, to be treated for what she was told by a practitioner was a cancerous tumor of the cheek. On examination, a tumor the size of a small chestnut was found with an ulceration of the mucous membrane, just filling the sharp edge of one fang of a carious molar tooth of the lower jaw, which was making its way from the gum. Being fully assured from former experience of many cases of a similar kind, that this was the sole cause of the tumor and ulceration, Mr. S. removed the tooth. The patient appeared again on the next clinic day, Dec. 17. The ulceration was healed, and the tumor gone.

Now I tell you, says Mr. Smith, if the cause of that tumor had been overlooked, no treatment of any kind would have been of the least avail, it would have continued, it would have increased, and gone on from bad to worse for months, and possibly for years, unless the tooth had been removed by nature

Several years ago, one of my own relatives died a most terrible death from a cancerous tumor of the cheek, located and aggravated by just such a ragged tooth.

It would seem impossible that a physician of any intelligence would overlook such a primary lesion, but in this particular case it certainly was overlooked, not only by one, but by many. Prof. Mütter, who operated on the case, was, as I have been informed, the first to point it out, but when, of course, it was too late.

More than thirty years ago (Braithwaite) Mr. Hey excised a malignant looking tumor from the tongue of a young country woman, a private patient of his; in a few days he was surprised to find that it had sprouted out as large, or larger than before the operation. Mr. Smith, who was called to examine the case, says: "On examining the tumor, which was dark, foul, and fungoid, and which bled at the slightest touch, giving great pain at every attempt to speak or masticate, I detected two broken incisors, the middle and left lateral leaning inward, and with sharp-pointed edges fitting into the center of the tumor. I was immediately convinced that these two teeth were the cause of all the mischief, and stated that opinion to Mr. Hey, who appeared doubtful. I said that he would not be justified in applying ligatures, or using any other means, without first waiting to see the effect of the removal of the two broken carious teeth. I never saw the young woman again, but was informed by Mr. Hey that the teeth were drawn, and that soon after the tumor disappeared, without any other means being resorted to.—*Medical & Surgical Reporter.*

